# Usefulness of C-reactive protein-triglyceride glucose index in detecting prevalent coronary heart disease: findings from the National Health and Nutrition Examination Survey 1999–2018

**DOI:** 10.3389/fcvm.2024.1485538

**Published:** 2024-10-15

**Authors:** Ming Xu, Lingyun Zhang, Dong Xu, Wenrui Shi, Weiguo Zhang

**Affiliations:** ^1^ Department of Cardiology, The People’s Hospital of Suzhou New District, Suzhou City, China; ^2^ Suzhou Medical College of Soochow University, Suzhou City, China; ^3^ Department of Cardiology, Shanghai Chest Hospital, Shanghai, China

**Keywords:** coronary heart disease, inflammation, insulin resistance, general population, epidemiology

## Abstract

**Background:**

Coronary heart disease (CHD) is one of the leading causes of mortality. The current study aims to assess the association between C-reactive protein-triglyceride glucose index (CTI) and the risk of prevalent CHD and to evaluate the usefulness of CTI to refine the identification of prevalent CHD.

**Methods:**

19,451 subjects from the National Health and Nutrition Examination Survey 1999–2010 were enrolled. CHD was ascertained according to the questionnaire.

**Results:**

The prevalent of CHD was 6.23%. After adjusting for conventional cardiovascular risk factors, each SD increase of CTI could cast a 1.357 times risk of CHD. In quartile analysis, the top quartile had a 1.807 times risk of CHD than the bottom quartile. Smooth curving fitting displayed that the association was linear in the entire range of CTI. Subgroup analysis revealed that the association was robust among several common subpopulations but stronger in subjects aged <60. Finally, both ROC and reclassification analysis demonstrated a significant improvement in identifying CHD when introducing CTI to the Framingham risk score.

**Conclusion:**

CTI has a positive, linear, and robust association with prevalent CHD in the general American population, and CTI may help to improve the detection of prevalent CHD in the general population.

## Introduction

Coronary heart disease (CHD) is a chronic cardiac disease caused by atherosclerotic lesions in coronary vessels; the development or rupture of the lesions will result in total occlusion of the coronary vessels, which is defined as myocardial infarction (1). In America, CHD maintained a high prevalence (around 6.0%–6.2%) in the past decade (2); and CHD also contributed substantially to the economic and health burden around the world (3, 4).

Chronic inflammation initiates and promotes atherosclerosis (5). Inflammation-induced endothelial dysfunction increases permeability to oxidized lipoproteins and their subendothelial accumulation, leukocyte recruitment, and platelet activation. During chronic inflammation, pro-inflammatory cytokines recruit macrophages to the vascular wall. Macrophages exert the catabolic role and thin the fibrous cap of the atherosclerotic foci, making the plaque unstable and prone to rupture and developing thrombosis (6, 7). Published articles have illustrated the association between inflammatory markers [C-reactive protein (CRP), leukocyte count, and albumin] and CHD (8–10). A recent investigation also suggests that the number of abnormal inflammatory markers was associated with an increased risk of CHD (11). However, the efficiency of inflammatory markers in detecting prevalent CHD is still low.

Insulin resistance (IR) also plays a critical role in the development of CHD (12). IR is associated with all components of metabolic syndrome, including dyslipidemia, hypertension, and central obesity; all these are risk factors for CHD (13). IR itself has also been identified as an independent risk factor of CHD (14, 15). On the contrary, an increment of insulin sensitivity has been revealed to lower the risk for CHD in a young population (16). Laboratory studies have illustrated that IR could promote CHD by promoting vascular stiffness, accelerating atherosclerotic plaque and thrombosis formation, and maintaining systemic inflammation (12, 17). Recently, non-invasive markers of IR, such as Triglycerides-glucose index (TyG), have been formulated to estimate IR (18). Its high correlation with invasively measured IR makes daily monitoring of IR possible (19). However, the value of TyG in detecting the prevalent CHD is still limited.

The recently proposed “C-reactive protein-triglyceride glucose index (CTI)” was formulated to comprehensively estimate both inflammation and IR severity (20). It has been shown to have significant value in diseases associated with inflammation and IR (20, 21). Therefore, the current analysis aims to evaluate the association between CTI and the prevalent CHD, and to explore the value of CTI in detecting the prevalent CHD in the general population.

## Methods

### Study participants

The datasets were originated from the National Health and Nutrition Examination Survey (NHANES). In brief, NHANES is a continuous survey hosted by the American National Center for Health Statistics. The survey adopted a cross-sectional design and was conducted every two years in the United States as a round. To maintain its representativeness, the survey employed a multistage, stratified, and clustered probability sampling design. Accordingly, data from different survey rounds could be integrated for analysis. Detailed information about NHANES, including recruitment procedures, population characteristics, and study design, can be found on the Centers for Disease Control and Prevention’s website (https://www.cdc.gov/nchs/nhanes/index.htm).

**Figure 1:**
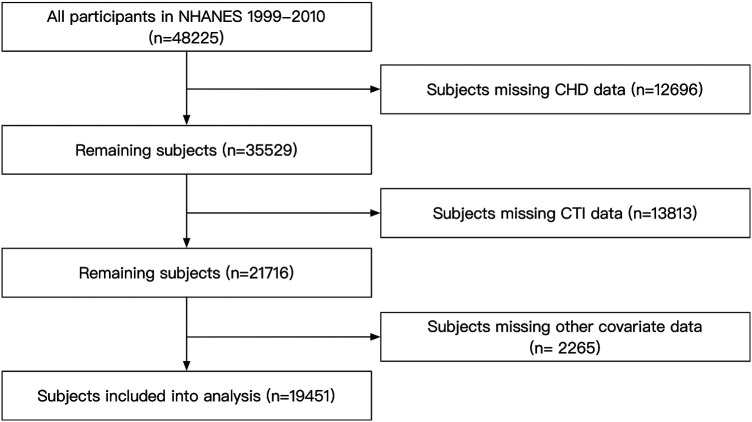
Flow chart of the subjects’ enrollment. NHANES, National Health and Nutrition Examination Survey; CTI, C-reactive protein-triglyceride glucose index; CHD, coronary heart disease.

In the current analysis, we enrolled subjects from NHANES 1999–2010. Subjects with missing data on CHD, CTI, and covariates were excluded. Finally, our study included 19,451 participants aged 20 to 85 years (Figure 1). The NCHS Institutional Ethics Review Board approved the protocol of the NHANES survey. Since our study contained no personally identifiable information, further ethical review was not required. All the data used in our study can be accessed through the official NHANES website.

### Data collection

The data collection process of the NHANES survey involved in-home interviews and subsequent laboratory tests. A computer-assisted system facilitated the in-home interviews. Demographic data and medical history data were recorded during the interview.

Anthropometric measurements were conducted according to a standardized procedure. Weight was measured to the nearest 0.1 kg while heights were measured to the nearest 0.1 cm. Waist circumference (WC) was measured at the horizontal level 1 cm above the umbilicus, and was recorded to the nearest 0.1 cm. After resting for more than five minutes of quiet sitting, blood pressure was measured. In the current study, the mean of three blood pressure readings was analyzed. The “Physician Examination Methods Manual” on the NHANES website provides further information regarding blood pressure measurement.

Laboratory tests were conducted at laboratories certified by the CDC. Blood lipids were quantified by enzymatic assay on the Roche Modular *P* and Roche Cobas 60,000 chemistry analyzer; Fasting plasma glucose (FPG) was measured by the oxygen rate method on the Modular Chemistry side of the Beckman DxC800; Serum creatinine (Scr) was measured using the Jaffe rate method by the DxC800 modular chemistry side; CRP was quantified by latex-enhanced nephelometry.

### Definition

Current drinking was defined as consuming alcohol at least twelve times in the year prior to enrollment. Current smoking was classified as reporting smoking cigarettes “some days” or “every day” in response to the question “Do you currently smoke cigarettes?”. The poverty-to-income ratio (PIR) was used to estimate the socioeconomic status of every subject; PIR was defined as the family income ratio to the federal poverty threshold. Body mass index (BMI) was calculated as weight (kg) ratio to height (m) squared. Participants were considered to receive anti-diabetic therapy if they reported taking medication to lower blood sugar levels or were currently using insulin; FPG ≥ 7 mmol/L and/or self-reported use of anti-diabetic therapy was defined as diabetes mellitus (DM) (22). Affirm answer to the question “Now taking prescribed medicine for hypertension” was regarded as anti-hypertensive therapy; hypertension was defined as a mean systolic blood pressure (SBP) ≥ 140 mmHg, and/or a mean diastolic blood pressure (DBP) ≥ 90 mmHg, and/or anti-hypertensive therapy (23). Coronary heart disease (CHD) history was deﬁned as answering “yes” to the question “Someone ever told you had coronary heart disease” or “Ever told you had a heart attack”. The Framingham risk score was formulated based on D’Agostino's work; the score was calculated based on age, total cholesterol (TC), high-density lipoprotein cholesterol (HDL-c), SBP, smoking status, and diabetes, and the score was calculated differently based on sex and anti-hypertensive therapy (24). TyG was formulated as ln [TG (mg/dl) × FPG (mg/dl)/2] (18). CTI was defined as 0.412*Ln (CRP) + ln [T.G. (mg/dl) × FPG (mg/dl)/2] (20).

### Statistical analysis

A statistical weighting was used in the current analysis to account for the survey design of NHANES. Categorical variables were summarized using frequencies and 95% confidence intervals (CI), while continuous variables were presented as mean values with corresponding 95% CIs. The chi-square test was used to compare categorical variables. *T*-test and Rank sum test were employed to compare normally distributed continuous variables and skewed distributed continuous variables. The main statistical analysis had two parts. In the first part, multivariate logistic regression analysis was conducted to estimate the association between CTI and prevalent CHD. CTI was first treated as a continuous variable. Then, CTI was divided into quartiles and analyzed as a categorical variable. In addition, a generalized additive model with a spline smooth-fitting function was used to explore the linearity of the association, and the linearity was tested via a logarithmic likelihood test. Finally, subgroup analysis was conducted to test whether the main result from the logistic regression was robust in conventional subpopulations. In the second part, receiver-operating characteristic curve (ROC) analysis and reclassification analysis were employed to assess the potential usefulness of CTI in improving the detection of prevalent CHD. The reclassification analysis included the continuous net reclassification index (NRI) and integrated discrimination index (IDI). All statistical analyses were performed in Stata Statistical Software (version 15.0; StataCorp. LLC., College Station, TX, USA), R (The R Foundation), and EmpowerStats (X&Y Solutions, Inc., Boston, MA, USA). A two-tailed *P*-value less than 0.05 was regarded as statistically significant.

## Results

The prevalence of CHD in the current study was 6.23% (1,211 of 19,451 subjects, Table 1). The mean age was statistically higher in the CHD group than in the non-CHD group. The percentage of males was also higher in the CHD group. Furthermore, the race distribution was also significantly different between groups; the CHD group had more non-Hispanic white subjects, while the non-CHD group had more Mexican American, non-Hispanic black, and other Hispanic subjects. The rates of current drinking and current smoking were slightly lower in the CHD group, but the differences were insignificant. PIR was significantly lower in the CHD group than in the non-CHD group. Regarding the measured parameters, weight, BMI, and WC were significantly lower in the non-CHD group. SBP was substantially higher in the CHD group. Even though DBP was significantly lower in the CHD group, the intergroup difference was relatively small. Data from the laboratory tests showed that FPG, triglycerides, Scr, and CRP were significantly higher in the CHD group, while TC and HDL-c were lower in the CHD group than in the non-CHD group. Results from the questionnaire displayed that the rates of anti-hypertensive and anti-diabetic therapy were significantly higher in the CHD group. Consistently, the prevalences of hypertension and diabetes were also higher in the CHD group than in the non-CHD group. Finally, both TyG and CTI were significantly higher in the CHD group than in the non-CHD group.

**Table 1:** Subjects’ characteristics.

Variables	Total (*n* = 19,451)	CHD (*n* = 1,211)	Non-CHD (*n* = 18,240)	*P* value
Age (years)	45.79 (45.31–46.28)	64.64 (63.77–65.51)	44.84 (44.38–45.29)	<0.001
Male (%)	48.68 (48.03–49.33)	65.49 (61.83–68.98)	47.82 (47.11–48.54)	<0.001
Race (%)				<0.001
Mexican American	7.57 (6.38–8.97)	2.90 (2.12–3.97)	7.81 (6.59–9.23)	
Other Hispanic	4.96 (3.73–6.57)	2.26 (1.33–3.83)	5.09 (3.84–6.73)	
Non-Hispanic white	72.54 (69.83–75.10)	81.80 (78.81–84.45)	72.07 (69.33–74.66)	
Non-Hispanic black	10.03 (8.60–11.67)	8.04 (6.54–9.83)	10.13 (8.67–11.80)	
Others	4.90 (4.25–5.64)	5.00 (3.41–7.26)	4.90 (4.24–5.65)	
Current drinking (%)	44.41 (42.24–46.60)	42.82 (37.93–47.86)	44.49 (42.35–46.65)	0.450
Current smoking (%)	20.30 (19.11–21.56)	18.87 (16.22–21.84)	20.38 (19.15–21.66)	0.307
PIR	3.06 (2.98–3.14)	2.78 (2.64–2.91)	3.07 (2.99–3.15)	<0.001
Weight (kg)	80.91 (80.42–81.40)	84.07 (82.64–85.51)	80.75 (80.26–81.24)	<0.001
Height (cm)	169.13 (168.93–169.33)	169.09 (168.40–169.77)	169.13 (168.93–169.34)	0.346
BMI (kg/m*2)	28.20 (28.03–28.36)	29.28 (28.88–29.67)	28.14 (27.97–28.31)	0.001
WC (cm)	96.90 (96.45–97.35)	103.85 (129.09–132.30)	96.55 (96.10–96.99)	<0.001
SBP (mmHg)	122.35 (121.86–122.84)	130.69 (129.09–132.30)	121.93 (121.45–122.40)	<0.001
DBP (mmHg)	71.79 (71.44–72.14)	69.20 (68.26–70.14)	71.92 (71.57–72.27)	<0.001
FPG (mmol/L)	5.32 (5.28–5.35)	6.07 (5.92–6.23)	5.28 (5.25–5.31)	<0.001
TC (mmol/L)	5.20 (5.17–5.22)	4.94 (4.84–5.04)	5.21 (5.18–5.24)	<0.001
TG (mmol/L)	1.69 (1.66–1.73)	1.93 (1.82–2.05)	1.68 (1.65–1.72)	<0.001
HDL-c (mmol/L)	1.385 (1.370–1.400)	1.285 (1.248–1.322)	1.390 (1.375–1.404)	<0.001
Scr (μmol/L)	77.01 (76.38–77.63)	93.23 (89.86–96.61)	76.18 (75.55–76.81)	<0.001
CRP (mg/dl)	0.41 (0.40–0.42)	0.53 (0.48–0.58)	0.40 (0.39–0.42)	<0.001
Anti-hypertension therapy (%)	21.98 (20.96–23.04)	60.23 (56.51–63.84)	20.04 (19.12–20.98)	<0.001
Anti-diabetic therapy (%)	5.75 (5.32–6.22)	19.78 (16.89–23.02)	5.04 (4.63–5.48)	<0.001
Hypertension (%)	30.69 (29.48–31.92)	68.14 (64.23–71.81)	28.78 (27.63–29.96)	<0.001
Diabetes (%)	8.39 (7.82–8.99)	25.03 (22.25–28.02)	7.54 (6.98–8.14)	<0.001
Framingham risk score (%)	3.20 (3.05–3.35)	10.83 (10.25–11.40)	2.81 (2.68–2.94)	<0.001
TyG	8.64 (8.62–8.66)	8.92 (8.87–8.97)	8.63 (8.60–8.64)	<0.001
CTI	7.94 (7.92–7.97)	8.37 (8.31–8.43)	7.92 (7.90–7.95)	<0.001

Data were summarized as mean (95% confidence intervals) or numbers (95% confidence intervals) according to their data type.

CHD, coronary heart disease; PIR, poverty-to-income ratio; BMI, body mass index; WC, waist circumference; SBP, systolic blood pressure; DBP, diastolic blood pressure; MBP, mean blood pressure; FPG, fasting plasma glucose; TC, total cholesterol; TG, triglycerides; HDL-c, high-density lipoprotein cholesterol; Scr, serum creatinine; CRP, C reactive protein; TyG, triglycerides-glucose index; CTI, C-reactive protein-triglyceride glucose index.

The association between CTI and the prevalent CHD was summarized in Table 2. In the non-adjusted model, each SD increase of CTI cast an additional 56.9% risk of the prevalent CHD. After adjustment of demographic variables (age, sex, race, current smoking and drinking, PIR), the risk increment for every SD increase of CTI reduced to 33.9%. Further adjustment of BMI, WC, FPG, TC, triglycerides, Scr, CRP, SBP, anti-hypertensive therapy, and anti-diabetic therapy, the risk increase for every SD increment diminished to 35.7%. In the quartile analysis, the top quartile had a 1.807 times risk of prevalent CHD than the bottom group after adjusting for all covariates. And we observed a linear trend towards a higher risk of prevalent CHD from the 1st to the 4th quartile (*P* for trend = 0.001).

**Table 2:** Association between CTI and prevalent CHD.

Variables	Odds ratio (95% CI)					
	Crude	*P* value	Model 1	*P* value	Model 2	*P* value
CTI (Per SD increase)	1.569 (1.485, 1.658)	<0.001	1.339 (1.245, 1.440)	<0.001	1.357 (1.147, 1.604)	0.001
Quartiles of CTI						
Quartile 1	Reference		Reference		Reference	
Quartile 2	2.185 (1.754, 2.722)	<0.001	1.350 (1.083, 1.683)	0.008	1.307 (1.053, 1.624)	0.016
Quartile 3	2.673 (2.126, 3.360)	<0.001	1.546 (1.206, 1.980)	0.001	1.423 (1.101, 1.839)	0.008
Quartile 4	3.954 (3.185, 4.907)	<0.001	2.184 (1.741, 2.741)	<0.001	1.807 (1.314, 2.484)	<0.001
*P* for trend		<0.001		<0.001		0.001

Crude: no adjustment.

Model 1: age, sex, race, current smoking, current drinking, PIR.

Model 2: Model 1+ BMI, WC, FPG, TC, triglycerides, Scr, CRP, SBP, anti-hypertensive therapy, anti-diabetic therapy.

CTI, C-reactive protein-triglyceride glucose index; CHD, coronary heart disease; CI, confidence interval; SD, standard deviation; PIR, poverty-to-income ratio; BMI, body mass index; WC, waist circumference; FPG, fasting plasma glucose; TC, total cholesterol; Scr, serum creatinine; CRP,C reactive protein; SBP, systolic blood pressure.

**Figure 2:**
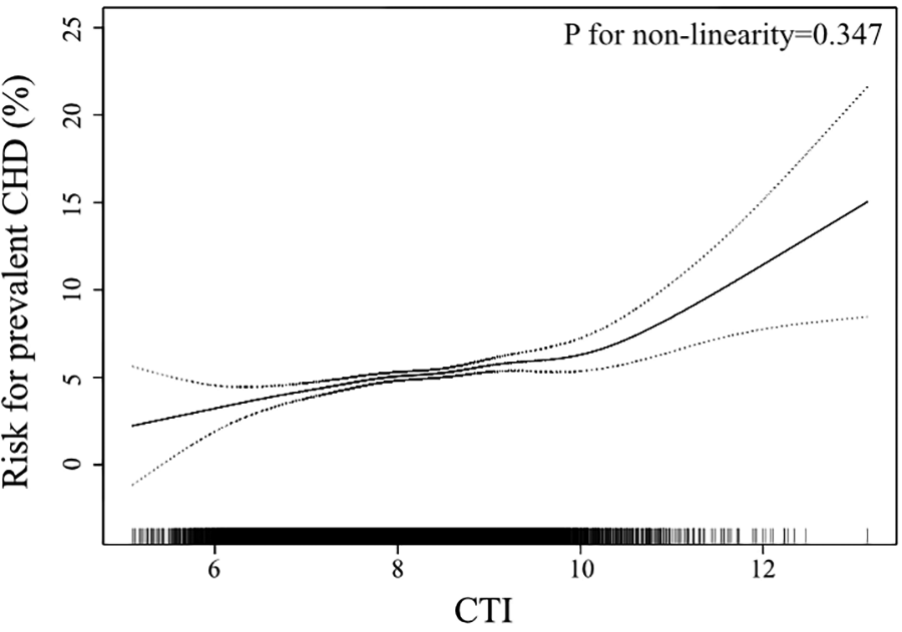
Smooth curve fitting to evaluate the linearity of the association between CTI and the prevalent CHD. The model was adjusted for age, sex, race, current smoking, current drinking, PIR, BMI, WC, FPG, TC, triglycerides, Scr, CRP, SBP, anti-hypertensive therapy, anti-diabetic therapy (The same as Model 2 in Table 2). The dotted lines depicted the pointwise 95% CI, and the continuous line showed the estimated risk of prevalent CHD. The association is linear in the whole range of CTI. CTI, C-reactive protein-triglyceride glucose index; CHD, coronary heart disease; PIR, poverty-to-income ratio; BMI, body mass index; WC, waist circumference; FPG, fasting plasma glucose; TC, total cholesterol; Scr, serum creatinine; CRP, C reactive protein; SBP, systolic blood pressure; CI, confidence interval.

In the smooth curve fitting analysis, we confirmed the linear trend observed in the quartile analysis (Figure 2). After adjustment of all covariates used in Model 2 of Table 2, the risk of prevalent CHD escalated linearly across the whole range of CTI. The logarithmic likelihood test confirmed the linearity was statistically significant (*P* for non-linearity = 0.347).

**Figure 3:**
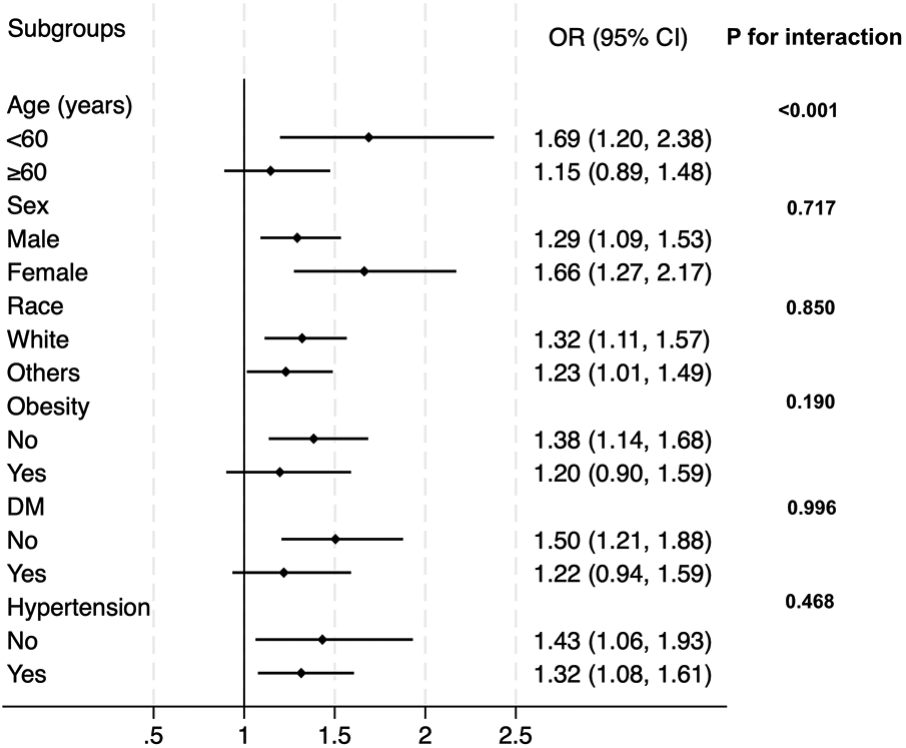
Subgroup analysis for the correlation between CTI and the prevalent CHD. The multivariate logistic model adjusted for all variables used in Model 2 of Table 2, except for the variable used to define subgroups. OR, odds ratio; DM, diabetes.

In the next step, we tested whether our main findings were robust in conventional subpopulations (Figure 3). The results demonstrated that the association between CTI and prevalent CHD was robust and similar in sex (male or female), race (white or others), obesity (yes or no), diabetes (yes or no), and hypertension (yes or no) subgroups. However, the association was interacted by age. In subjects aged less than 60, the OR value was relatively higher than that in the general population, while in subjects aged ≥60, the association was weaker than that in the general population. The *P* value for interaction was less than 0.001.

ROC and reclassification analysis were employed to test whether CTI could improve the detection of prevalent CHD in the general population (Table 3). In the ROC analysis, we observed that the AUC of CTI (0.610, 95% CI: 0.603–0.617) was significantly higher than that of CRP (0.562, 95% CI: 0.555–0.569) or TyG (0.587, 95% CI: 0.579–0.595). Then, CTI was combined with the Framingham risk score, and we observed a significant but marginal improvement in the CHD-detecting ability of the whole model (AUC: 0.844 vs. 0.846, *P* for comparison = 0.033). In the reclassification analysis, both continuous NRI (0.149, 95% CI: 0.091–0.207, *P* < 0.001) and IDI (0.002, 95% CI: 0.000–0.003, *P* = 0.025) also implicated the significant value of CTI to refine the identification of prevalent CHD in the general population.

**Table 3:** ROC and reclassification analysis of CTI on prevalent CHD identification.

Model	AUC (95% CI)	*P* value	*P* for comparison	NRI (continuous)	*P* value	IDI	*P* value
CTI	0.610 (0.603, 0.617)	<0.001	–	–	–	–	–
CRP	0.562 (0.555, 0.569)	<0.001	<0.001				
TyG	0.587 (0.579, 0.595)	<0.001	<0.001				
Framingham risk score	0.844 (0.839–0.849)	<0.001	–	–	–	–	–
Framingham risk score + CTI	0.846 (0.841–0.851)	<0.001	0.033	0.149 (0.091–0.207)	<0.001	0.002 (0.000, 0.003)	0.025

ROC, receiver operating characteristic curve; CTI, C-reactive protein-triglyceride glucose index; CHD, coronary heart disease; AUC, area under the curve; NRI, net reclassification index; IDI, integrated discrimination index; CRP, C reactive protein; TyG, triglycerides-glucose index.

## Discussion

In the current secondary analysis of the NHANES database, our data demonstrated a positive, significant association between CTI and the risk of prevalent CHD in the general population. The association was linear in the whole range of CTI, suggesting CTI could serve as a linear indicator of prevalent CHD risk. Moreover, the association was robust in sex, race, obesity, diabetes, and hypertension subgroups. The effect value of the association was higher in those aged <60 years but lower in subjects aged ≥60 years. Finally, our results also implicated the potential incremental value of CTI in identifying the prevalent CHD in the general population.

The findings from the current study confirmed our assumption. With the adjustment of demographic, anthropometric, laboratory, and medical history covariates, CTI still had a positive and significant association with the prevalent CHD. Notably, the association was independent of CRP, TG, and FPG, which are used to formulate CTI. Moreover, the risk of prevalent CHD increased proportionally with the increment of CTI, implicating that CTI could act as a linear index of the risk of prevalent CHD in the general population. The subgroup analysis observed a significant interaction between age and the association between CTI and the prevalent CHD. The OR of the association was significantly higher in subjects aged less than 60 years than in those aged ≥60 years. This phenomenon indicated that the association between CTI and the prevalent CHD was stronger in populations aged <60 years. The general practitioner should be more careful about the risk of potential existing CHD if a person less than 60 years old has an elevated CTI level. In subjects aged ≥60 years, the association between CTI and the prevalent CHD still existed. Accordingly, their risk of prevalent CHD still increased along with the increase of CTI, but the risk-increasing rate was not as high as those aged less than 60 years. In the sex, race, obesity, diabetes, and hypertension subgroups, no interaction effect was detected, and the effect values of the association in these subgroups were consistent with the OR in the whole population. Therefore, applying our findings directly to these populations should be reasonable. General practitioners do not need to pay additional attention to these subpopulations.

Based on the ROC and reclassification analysis results, CTI could improve the detection of prevalent CHD in the general population. Although the AUC of CTI alone was limited, the ROC results showed that the CTI had a larger AUC than TyG and CRP. Additionally, we observed a significant improvement when adding CTI into Framingham risk score (*P* for comparison = 0.033), suggesting the incremental value of CTI to refine the detection of prevalent CHD. However, although ROC analysis is the most common method to evaluate the value of a novel marker, it still has its limitations. ROC analysis compares the diagnostic ability of different models rather than assessing the effect of a novel marker to optimize the diagnostic ability of the whole model (25). Thus, ROC analysis could have a low sensitivity in identifying the value of a novel index to refine the detection of prevalent diseases (26). To address the drawback of ROC analysis, statisticians proposed reclassification analysis, aiming to confirm the improvement from novel indexes for optimizing the detection of prevalent diseases (27–29). Compared with ROC analysis, reclassification analysis focused on the incremental value of a novel biomarker for diagnosing or predicting diseases rather than the ability of the whole diagnosis or prediction model. Therefore, reclassification analysis could specifically test the diagnostic or predictive value of the novel biomarkers. However, reclassification analysis also has its limitations. First, it could not compare the diagnostic or predictive value of the two models. Therefore, the readers could not acquire the overall improvement of the diagnostic or predictive value of the new model containing the novel biomarker. Second, reclassification analysis is rarely used in studies, and the basic model used in different studies is variant. Therefore, comparing NRI and IDI of different biomarkers from different studies is impractical. Accordingly, the significance of NRI and IDI is more important than their values. Third, the reclassification analysis has a relatively higher sensitivity than the ROC analysis. Hence, some biomarkers could be overestimated by reclassification analysis. Overall, ROC and reclassification analysis evaluate a novel biomarker from different angles. The two analyses have their advantages and disadvantages. Since they are complementary, the results of the two analyses should be discussed together. In our work, both continuous NRI and IDI confirmed the significant improvement from CTI to improve the detection of prevalent CHD. In summary, both ROC and reclassification analysis implicate the potential value of CTI to optimize the detection of prevalent CHD in the general population.

The underlying pathophysiological mechanisms between CTI and CHD were inflammation and IR. Inflammation plays its role in several critical time points during the development of CHD. First, inflammation promotes the development of coronary atherosclerotic plaque. During chronic inflammation, excessive low-density lipoprotein cholesterol (LDL-c) is oxidized by reactive oxygen species and becomes oxidized LDL-c; the latter would accumulate in the middle-sized arterial wall, typically the coronary arterial wall. Monocytes would be recruited by inflammatory cytokines to these arterial walls, differentiate into macrophages and engulf oxidized LDL-c (30). Then, the macrophages would derive into lipid-rich foam cells, which are the fundamental part of the atherosclerotic core (31). Second, Inflammation also triggers the formation of calcifications within the necrotic lesion as part of the healing response to the inflammatory activation of macrophages (32, 33). The demise of macrophages and smooth muscle cells leads to the release of vesicles that serve as seeding sites for hydroxyapatite crystal deposition. These crystals can cluster together, forming microcalcifications smaller than 50 µm in diameter that become embedded in the fibrous cap (34, 35). Plaque calcification further promotes macrophage infiltration, increasing nucleating sites and additional calcification (36). If inflammation continues, it will result in repeated cycles of monocyte infiltration. These monocytes differentiate into macrophages, eventually dying, leading to microcalcification development (37). Third, inflammation is also a critical promotor of vulnerable plaques (38). Atherosclerotic plaque contains a large amount of extracellular matrix (ECM), including collagen, elastin, proteoglycan, and glycosaminoglycan, which is synthesized by smooth muscle cells in the arterial wall. Under inflammatory conditions, cytokines (IL-1β, TNF-α) trigger the secretion of metalloproteinases (MMPs), particularly MMP-1, MMP-8, MMP-9, MMP-12, and MMP-13, from macrophages, regulated by microRNAs (39, 40). MMPs facilitate the degradation of the extracellular matrix (ECM), leading to the thinning and weakening of the fibrous cap; this compromises the cap’s tensile strength, rendering the plaque unstable (41). IR also plays a vital role in the development and progression of CHD. As a core mechanism, insulin resistance connects all elements of metabolic syndrome (hypertension, dyslipidemia, hyperglycemia, and central obesity), which are significant risk factors for cardiovascular events (13). Furthermore, IR reduces glucose uptake and utilization in cardiomyocytes; the change in metabolism causes the heart to increasingly depend on fatty acid oxidation to provide energy, resulting in a modified preference for substrates and a higher demand for oxygen supply (42). Therefore, cardiomyocytes would be more vulnerable to ischemia. Moreover, IR and the subsequent hyperglycemia could cause injuries to the endothelium through multiple mechanisms, facilitating the formation of atherosclerosis (12). Additionally, IR could promote persistent inflammation, thereby promoting vascular stiffness via multiple mechanisms (17). Overall, published laboratory studies support the association between CTI and CHD.

Our study has several limitations that should be acknowledged. First, the cross-sectional design of NHANES precludes the determination of causal relationships between CTI and CHD. Therefore, we were unable to assess the predictive efficacy of CTI for CHD incidence in this study. Future research employing longitudinal designs is warranted to investigate these associations further. Second, our study excluded NHANES participants lacking relevant variables, potentially introducing selection bias to our results. Third, reliance on self-reported information in NHANES raises concerns about recall limitations and subjectivity, which may affect data accuracy. Future studies using more reliable definitions of CHD are needed to confirm our findings. Fourth, since NHANES was conducted exclusively in the United States, caution is required in generalizing our findings to other populations. Therefore, additional studies involving diverse populations are necessary to validate our results. Fifth, although the ROC analysis confirmed the statistical improvement from CTI to detect prevalent CHD, the increase in AUC value was still marginal. However, since both ROC and reclassification analysis support the value of CTI in detecting prevalent CHD, we believe the practical value of CTI still deserves more studies to evaluate. Lastly, although our analysis adjusted for several covariates, the potential influence of unmeasured confounders cannot be ruled out. Future studies incorporating more comprehensive data collection methods are essential to validate our findings.

## Data Availability

The datasets presented in this study can be found in online repositories. The names of the repository/repositories and accession number(s) can be found below: https://wwwn.cdc.gov/nchs/nhanes/ContinuousNhanes/Default.aspx?BeginYear=1999.
